# Draft Genome Sequence of *Magnetovibrio blakemorei* Strain MV-1, a Marine Vibrioid Magnetotactic Bacterium 

**DOI:** 10.1128/genomeA.01330-16

**Published:** 2016-11-23

**Authors:** Denis Trubitsyn, Fernanda Abreu, F. Bruce Ward, Todd Taylor, Masahira Hattori, Shinji Kondo, Urmi Trivedi, Sarah Staniland, Ulysses Lins, Dennis A. Bazylinski

**Affiliations:** a Southwestern Oklahoma State University, Weatherford, Oklahoma, USA; b Universidade Federal do Rio de Janeiro, Rio de Janeiro, Brazil; c University of Edinburgh, Edinburgh, United Kingdom; d RIKEN Center for Integrative Medical Sciences, Yokohama, Kanagawa, Japan; e Waseda University, Tokyo, Japan; f National Institute of Polar Research, Tokyo, Japan; g University of Sheffield, Sheffield, United Kingdom; h University of Nevada at Las Vegas, Las Vegas, Nevada, USA

## Abstract

We report here the genome sequence of *Magnetovibrio blakemorei* MV-1, a marine vibrioid magnetotactic bacterium with a single polar flagellum. The current assembly consists of 91 contigs with a combined size of 3,638,804 bp (54.3% G+C content). This genome allows for further investigations of the molecular biomineralization mechanisms of magnetosome formation.

## GENOME ANNOUNCEMENT

Magnetotactic bacteria are a heterologous group of prokaryotes that biomineralize nanosized, intracellular magnetic crystals surrounded by a phospholipid bilayer. These structures, termed magnetosomes, consist of magnetite (Fe_3_O_4_) or greigite (Fe_3_S_4_) and are generally organized in chains that cause cells to align along the earth’s geomagnetic field lines. This feature appears to be advantageous to motile aquatic bacteria as it reduces the need to navigate in three-dimensional space to movement in a single dimension in the search for optimal environmental conditions (usually microaerobic or anaerobic zones). Magnetosomes are not only excellent examples of prokaryotic biomineralization, but they also possess unique properties that are important for many biotechnological and medical applications (1).

*Magnetovibrio blakemorei* MV-1 is a marine magnetotactic vibrio with a single polar flagellum that is significantly different from other magnetotactic bacteria in its metabolism and in the morphology of its magnetosome crystals. Specifically, *M. blakemorei* MV-1 grows microaerophilically and anaerobically using nitrous oxide (N_2_O) as a terminal electron acceptor. It produces chains of elongated magnetosomes comprising magnetite with a crystal morphology described as truncated hexa-octahedrons (2).

The 3,638,804-bp *M. blakemorei* MV-1 genome sequence was generated by combining the following sequencing methods: Solexa (8× coverage), 454 (65× coverage), and Sanger sequencing (5,244 reads). Combining the assemblies of Solexa and 454 data resulted in 191 contigs. The assembly was improved by joining fragments using inverse and conventional PCR followed by Sanger sequencing of amplified regions. This approach reduced the number of contigs to 91. Automated annotation by RAST (3) generated 3,564 predicted coding sequences, including 1,157 hypothetical proteins and 49 total RNAs. The difficulty in sealing this genome into a single contig is related to the presence of 62 transposase genes.

Currently, there are no specific details on which genes determine the elongated morphology of magnetosome crystals. To determine this, genes have to be inactivated, which requires a tractable genetic system. The sequencing of the *M. blakemorei* MV-1 genome should provide information on the metabolic pathways to allow the development of such a genetic system. Specifically, evidence for the potential utilization of specific terminal electron acceptors for growth is present as genes within the genome identified through annotation. Several of these reductase genes were identified, including arsenate, dimethyl sulfoxide, sulfite, nitrate, nitrite, nitric oxide, and nitrous oxide, although the ability to support growth of some of these compounds needs to be tested. In addition, magnetosome synthesis requires an efficient iron acquisition system. The genome of *M. blakemorei* MV-1 contains the following iron transport genes: a ferric iron ABC transporter and permease, a ferric siderophore transport system, ferrous iron transporters, a high-affinity Fe^2+^/Pb^2+^ permease, and a high-affinity iron transporter.

Finally, access to the *M. blakemorei* MV-1 genome will allow for transcriptome analysis to investigate novel magnetosome-related genes not previously described in other magnetotactic bacteria that are expressed during magnetosome synthesis.

### Accession number(s).

This whole-genome shotgun project has been deposited at DDBJ/ENA/GenBank under the accession number MCGG00000000. The version described in this paper is the first version, MCGG01000000.
